# Low prevalence of *Schistosoma haematobium* infection in pregnant women in Buffalo City district

**DOI:** 10.4102/sajid.v38i1.521

**Published:** 2023-06-15

**Authors:** Remco P.H. Peters, Mandisa Mdingi, Hyunsul Jung, Freedom Mukomana, Ranjana M.S. Gigi, Andrew Medina-Marino, Jeffrey D. Klausner

**Affiliations:** 1 Research Unit, Foundation for Professional Development, East London, South Africa; 2 Department of Medical Microbiology, Faculty of Health Sciences, University of Pretoria, Pretoria, South Africa; 3 Division of Medical Microbiology, University of Cape Town, Cape Town, South Africa; 4 Department of Social and Preventive Medicine, University of Bern, Bern, Switzerland; 5 Graduate School for Cellular and Biomedical Sciences, University of Bern, Bern, Switzerland; 6 Desmond Tutu HIV Centre, University of Cape Town, Cape Town, South Africa; 7 Department of Psychiatry, Perelman School of Medicine, University of Pennsylvania, Pennsylvania, United States of America; 8 Keck School of Medicine, University of Southern California, Los Angeles, United States of America

To the Editor,

Adverse pregnancy outcomes such as stillbirth, pre-term birth and low birth weight are common in South Africa. The aetiology of these conditions is multifactorial and infections play an important role. Studies have shown an increased risk of adverse pregnancy outcomes associated with sexually transmitted infection (STI) during pregnancy.^1^ Urogenital *Schistosoma haematobium* is another infection that should be considered for adverse pregnancy outcomes.^2^

Schistosomiasis, also known as bilharzia, is a parasitic infection acquired through contact with contaminated surface water. Adult worms mainly live in the venous plexus surrounding the bladder and genital tissue, depositing eggs in the urogenital tract. Female genital schistosomiasis may present as genital burning, vaginal discharge and pain and has an increased risk of HIV infection.^3^ In pregnancy, schistosomiasis has been associated with anaemia, miscarriage, stillbirth, pre-term delivery and low birth weight, although infection intensity may play a role.^4,5,6^

Schistosomiasis prevalence studies are generally conducted in schoolchildren in whom haematuria is an important manifestation. In contrast, there is a paucity of epidemiological data of schistosomiasis in pregnant women despite the health implications for mother and foetus.^2^ We conducted a prevalence study of *S. haematobium* infection in pregnant women in the Buffalo City Metropolitan (BCM) health district, Eastern Cape province, South Africa.

Adult women (≥ 18 years) were enrolled within an ongoing implementation-effectiveness trial of the effects of STI screening on pregnancy outcomes at four healthcare facilities between March 2021 and October 2022.^7^ These facilities represent good geographic coverage of the BCM district. In brief, women were recruited and consented at their first antenatal care visit. Study activities included: administering of a questionnaire, a physical examination, and on-site STI testing. Midstream urine specimens were collected for urinalysis using dipstick (LifeSADX, Houston, United States) and shipped to the Department of Medical Microbiology at the University of Pretoria for microscopy. After centrifugation (3000 g; 5 min) of the urine sample, *S. haematobium* eggs were quantified using light microscopy (40x); observation of ≥ 1 egg was classified a positive read. The slide microscopy method has suboptimal sensitivity when compared with antigen and molecular detection; however, those tests were not available to the team.

Microscopy was performed of urine samples from 966 pregnant women. Median age was 28 years (range: 18–44 years), median gestational age at enrolment was 13.7 weeks (range: 2.4 to 26.8 weeks) and 287 women (29.7%) were living with HIV. Water contact with a stream, river or lake was reported by 104 participants (11%): frequent crossings by 75 (7.8%), doing laundry 18 (1.9%), collecting water 8 (9.8%), bathing 5 (0.5%) and fishing 1 (0.1%). Urine dipstick was positive for erythrocytes in 23 women (2.4%); these were diagnosed with urinary tract infection with or without STIs.

**FIGURE 1:**
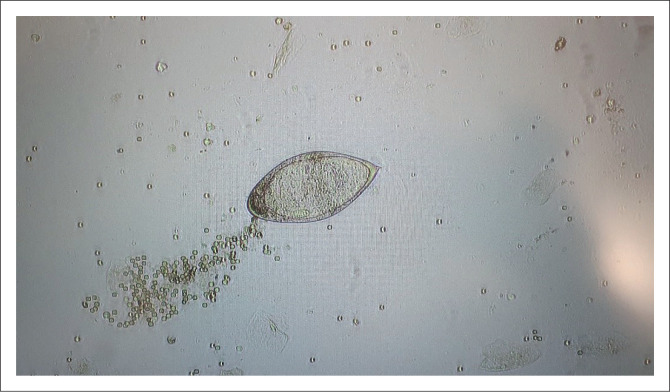
Microscopy image showing *Schistosoma haematobium* egg in a wet mount of urine concentrate from a pregnant woman.

Microscopy was positive for *S. haematobium* (Figure 1) in only one urine specimen (0.1%; 95% confidence interval: 0.0% – 0.3%). This pregnant woman (7 weeks; 0 days) was living in an informal settlement, used a community tap for drinking water and reported frequent water crossing. She did not report travelling to other districts in recent years. No symptoms were reported, she tested negative for HIV, and the urine dipstick result was normal. The participant was treated with praziquantel and the notifiable disease case notification form was completed. She delivered a healthy baby.

Schistosomiasis is endemic in the Eastern Cape province with an estimated prevalence of 141 per 100 000,^8^ but regional differences are present and changes over time may occur.^9^ We only diagnosed one pregnant woman with *S. haematobium* infection (prevalence estimate of 0.1%) suggesting that prevalence in the BCM district is low. Most likely, prevalence in pregnant women is higher further northeast in the province, for example, 73% of schoolchildren in Mbashe tested positive for *S. haematobium* using the ‘Transkei slide’ microscopy.^10^ However, the relationship between prevalence of schistosomiasis in schoolchildren and prevalence in pregnant women in the same setting is unclear given that we identified a case of schistosomiasis that may have been locally acquired; a survey of schoolchildren in the same area could be useful. In the absence of data, epidemiological studies of schistosomiasis in pregnancy are warranted in the endemic areas of the Eastern Cape as well as the Limpopo, Mpumalanga, and KwaZulu-Natal provinces.^8^

The low prevalence of *S. haematobium* in pregnancy should not discourage efforts to address the burden of schistosomiasis in pregnancy in other endemic regions of Southern Africa. Given the health implications of untreated infection and the multifactorial aetiology of adverse pregnancy outcomes, global efforts to improve pregnancy outcomes should continue to include schistosomiasis management and control.
